# Lung development and regeneration: newly defined cell types and progenitor status

**DOI:** 10.1186/s13619-022-00149-0

**Published:** 2023-04-03

**Authors:** Xiaogao Meng, Guizhong Cui, Guangdun Peng

**Affiliations:** 1 Center for Cell Lineage and Development, Guangzhou Institutes of Biomedicine and Health, Chinese Academy of Sciences, Guangzhou, 510530 Guangdong China; 2 Life Science and Medicine, University of Science and Technology of China, Hefei, 230026 Anhui China; 3 School of Basic Medical Sciences, Guangzhou Laboratory, Guangzhou Medical University, Guangzhou, 510005 China

**Keywords:** Lung development, Regeneration, Stem cell, Progenitor, scRNA-seq, Lineage tracing

## Abstract

The lung is the most critical organ of the respiratory system supporting gas exchange. Constant interaction with the external environment makes the lung vulnerable to injury. Thus, a deeper understanding of cellular and molecular processes underlying lung development programs and evaluation of progenitor status within the lung is an essential part of lung regenerative medicine. In this review, we aim to discuss the current understanding of lung development process and regenerative capability. We highlight the advances brought by multi-omics approaches, single-cell transcriptome, in particular, that can help us further dissect the cellular player and molecular signaling underlying those processes.

## Background

The lung is a complex organ consisting of multiple cell lineages, including epithelial cells, which are the most abundant and diverse cell types lining the luminal surface of the airway trees and alveoli, and various stromal cells, endothelial cells, blood, and immune cells supporting the pulmonary parenchyma (Travaglini et al. 2020). The essential function of the lung is to exchange oxygen in the air with carbon dioxide in the blood. At the same time, constant exposure to the external environment makes the lung more vulnerable, leading to infection, injury, and lung diseases. For example, the infection of severe acute respiratory syndrome coronavirus 2 (SARS-CoV-2) causes coronavirus disease 2019 (COVID-19) (Jain et al. 2020; Malik 2020, Rehman et al. 2020; Xiao et al. 2020), which results in the damage of lung alveoli and subsequent severe pneumonia or even disease of life-threatening (Azkur et al. 2020; Coperchini et al. 2020; Soy et al. 2020). Respiratory diseases are the leading causes of disability and death globally, widespread both in adults and infants. For instance, bronchopulmonary dysplasia (BPD) and neonatal respiratory distress syndrome (RDS) are prevalent in premature neonates and idiopathic pulmonary fibrosis (IPF), chronic obstructive pulmonary disease (COPD), asthma, cystic fibrosis and acute respiratory distress syndrome (ARDS) in adults. Statistically, more than 7 million deaths are attributed to lung disease annually (Gibson et al. 2013), accounting for every sixth death globally (Schiller et al. 2019), mainly resulting from irreversible damage of lung injury and the inability to demand lung transplantation. Besides, lung transplantation is a high-risk surgery, and its five-year survival is only 59%, according to recent data (Bos et al. 2020). Thus, the application of stem cells residing in the adult lung for repair after lung injury is an important part of regenerative medicine, and some excellent review papers are talking about those topics (Wu and Tang 2021; Zepp and Morrisey 2019). Recent studies have also found that various unappreciated progenitor cells reside in the developing lung, pointing to potential therapeutic implications. Therefore, querying cell atlas in single-cell and spatial contexts during lung development and regeneration may empower new strategies to re-engineer functional cells. In this review, we summarize the current findings about lung development progress and stem cells present in adult lungs and discuss their therapeutic potential for lung regeneration.

## Lung structure and cellular compositions

### Epithelial cells in proximal airways

**Fig. 1:**
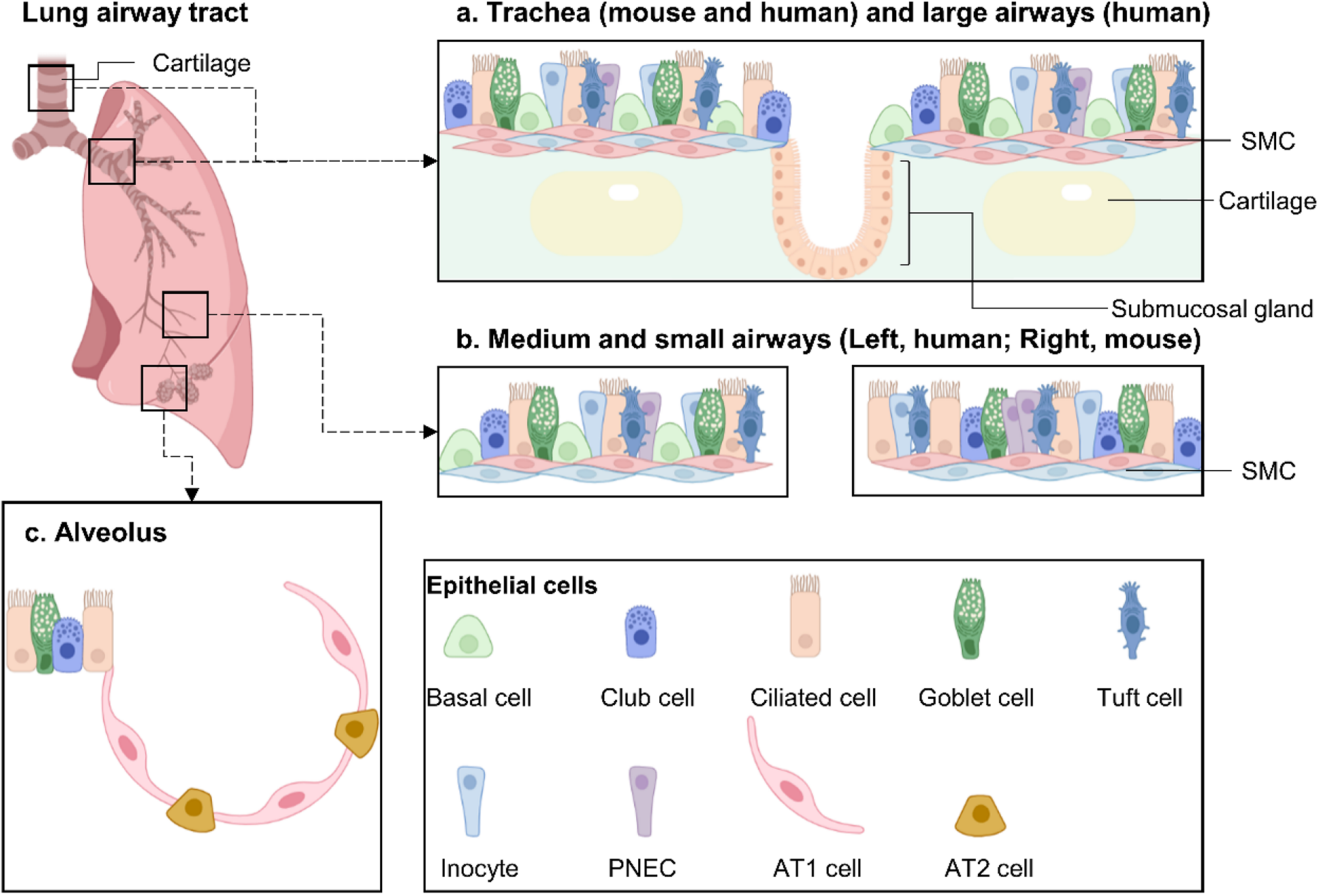
The anatomy and cellular composition in mammalian lung. **a**, **b** The architecture of epithelial cells within mouse and human airways. The basal cells, club cells, ciliated cells, goblet cells, tuft cells, pulmonary neuroendocrine cells and pulmonary ionocytes are observed in the proximal airways both in human and mouse. Basal cells are resident stem cells for trachea and main bronchi in mouse lung and trachea and proximal airways in human lung. **b** Mouse small airways don’t contain basal cells comparing to human **c** Cellular composition of alveoli. The alveoli include the alveolar type 1 epithelial cells (AT1 cells) and AT2 cells

The proximal region of the lung starts from the trachea, which reaches out in left and right direction to generate large and small branching airways and terminate in the alveoli. The proximal airways, both in humans and mice, consist of various cell types, including basal cells, club cells, ciliated cells, goblet cells, tuft cells (brush cells), pulmonary neuroendocrine cells (PNECs), and pulmonary ionocytes (Fig. 1a, b), which has been cataloged by single-cell RNA sequencing (scRNA-seq) recently (Montoro et al. 2018; Plasschaert et al. 2018). Club cells and goblet cells can produce mucus to trap inhaled particulates and microorganisms, which are the critical first line of immune defense of the lung. Ciliated cells have cilia on their apical surface, shuttling the particulates trapped by mucus out of the airway in a retrograde way. Basal cells are resident stem cells for the trachea and main bronchi in mouse lung and trachea and proximal airways in human lung, capable of repopulating other cell lineages during homeostasis and regeneration. Different from other epithelium, PNECs are innerved and could function as sensory cells within the airways. Recently, with the application of scRNA-seq, some rare airway lineages in PNECs have been characterized. For example, secretory granules loaded with neurotransmitters, including calcitonin-gene-related peptide and γ-aminobutyric acid, were found in PNECs (Barrios et al. 2019; Song et al. 2012) to bridge environmental sensation to neuro-peptide and neurotransmitter outputs (Branchfield et al. 2016). PNECs have also been found to regulate immune response to allergens and critically influence mucus production (Barrios et al. 2019; Sui et al. 2018).

The cellular composition and hierarchy of airway tracheal epithelium has been delineated further by scRNA-seq technology (Montoro et al. 2018; Plasschaert et al. 2018). Unlike residing in other mucosal tissues, such as the intestine, where they act as an important first innate immune line defending against parasite infection, producing cytokines like IL-25 (Gerbe and Jay 2016; von Moltke et al. 2016), tuft cells in airways have a microtubule network which extends to apical microvilli on the airway surface and expresses a plenty of sensory function related G protein-coupled receptors and taste receptors (Krasteva and Kummer 2012; Montoro et al. 2018), besides being involved in asthmatic inflammation.

Another rare cell type lining the adult murine and human tracheal and proximal airways, termed pulmonary ionocytes, were identified by two independent studies (Montoro et al. 2018; Plasschaert et al. 2018). This kind of cell specifically expresses high levels of cystic fibrosis transmembrane conductance regulator gene (*CFTR*) transcripts and originates from the basal cell (Montoro et al. 2018; Plasschaert et al. 2018). CFTR mutation will cause cystic fibrosis and accompanying alterations of the mucus viscosity lining the airway, then lead to infection predisposition (Ratjen et al. 2015). These findings suggest that this kind of cell may play a crucial cellular role in cystic fibrosis.

In addition, there are also cartilaginous rings and submucosal glands underlining the trachea in the mouse and the tracheal region and large airways in the human airway tract. The submucosal glands contain mucous cells, serous cells, and myoepithelial cells, which are with goblet cells together, responsible for luminal mucus production. Leveraging the power of single-cell RNA-seq, we keep our understanding of the cellular ecosystem within the lung witnesses constantly updating.

### Two types of alveolar epithelial cells

The alveoli are different from proximal airways in both anatomical and cellular compositions. There are two major epithelial cells in alveoli: one is alveolar type 1 epithelial cells (AT1 cells), and the other is alveolar type 2 epithelial cells (AT2 cells) (Fig. 1c). AT1 cells are flattened and squamous, and they cover more than 95% of the gas exchange surface of the alveoli and are closely associated with the surrounding endothelial capillary plexus to form the thin gas diffusion surface. AT2 cells are cuboidal and secret pulmonary surfactant, which could reduce the surface tension of the alveolus to avoid collapsing during ventilation. Of note, AT2 cells also act as progenitors in alveoli, which can self-renew and differentiate into AT1 cells during lung development and regeneration (Barkauskas et al. 2013; Frank et al. 2016). The synthesized attributes of the alveolus entail a complex array of cell lineages, such as endothelial cells, alveolar macrophages, and mesenchymal cells.

## Overview of the lung development process

The lung, as well as the trachea, originates from the anterior foregut endoderm, a tissue that generates various internal organs, including the respiratory system, esophagus, thyroid, and liver. The specification of the lung starts from embryonic day (E)9.0 in mice and gestation week (W)4-5 in humans when the transcriptional factor *Nkx2-1* expresses in the endoderm cells on the ventral side of the anterior foregut. Lung development is an elegant and complicated process composed of 5 different developmental stages among mammals which are embryonic, pseudoglandular, canalicular, saccular, and alveolarization stages. The whole process of the lung development consists of two critical developmental events: the formation of conducting airways, termed branching morphogenesis, and functionalization of the alveolar region, referred to alveolarization. And there are some excellent reviews about this topic which we encourage readers who are interested to refer to (Herriges and Morrisey 2014; Nikolić et al. 2018; Schittny 2017; Zepp and Morrisey 2019).

### Progenitors and cell lineages in the developmental lung

**Fig. 2:**
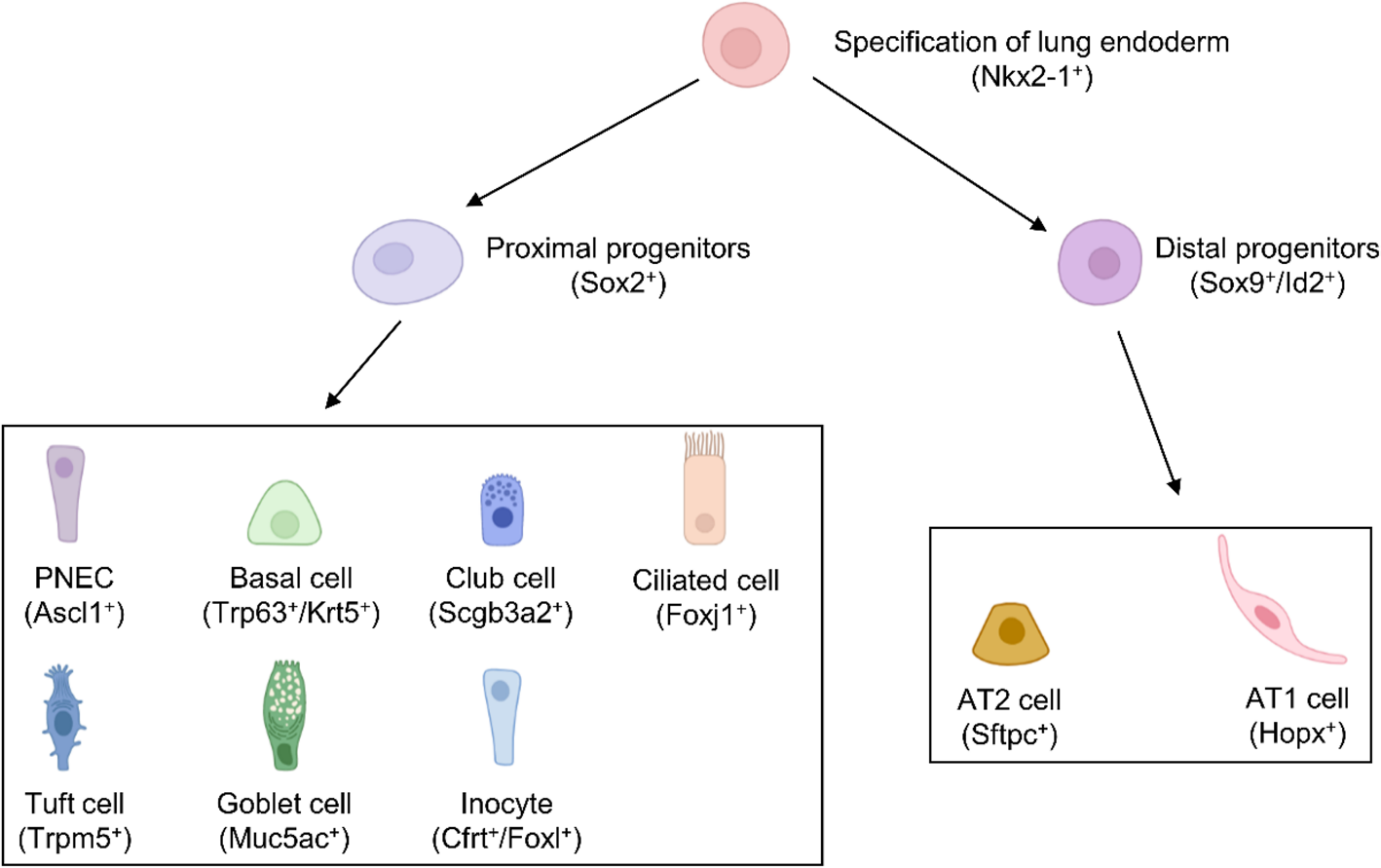
Cell lineages within the developing lung. The Nkx2-1-positive endoderm developed into both Sox2 positive proximal progenitors and Sox9 and Id2 double positive distal progenitors. The proximal and distal progenitors give rise to distinct sets of differentiated cells. Markers of each cell type are indicated in parentheses

In mice, the formation of proximal–distal airway pattern is defined by SOX2 expression in the proximal airway epithelium, and SOX9 and ID2 expression in the distal tip (Fig. 2), which will give rise to proximal and distal epithelial cell lineage respectively. By contrast, SOX2 and SOX9 are both expressed in the distal tips of human developing lungs (Waghray and Rajagopal 2017).

The cellular differentiation of mouse airway epithelium mostly occurs during lung branching morphogenesis until E16.5. Although the appearance of different airway epithelial cell lineages could not be easily discerned by the morphology during the early lung development stage, their presence could be detected by the expression of distinct marker genes. As indicated in a study in mice, TRP63^+^ basal cells are differentiated as early as E10.5. These cells are multipotent and able to convert to both proximal and distal epithelial cell fates (Yang et al. 2018). Club cells, marked by *Scgb3a2*, emerge at E11.5 in the developmental mouse lung (Guha et al. 2012; Kurotani et al. 2008), whereas, the existence of ciliated cells marked by FOXJ1 expression is observed by E14.5 (Rawlins et al. 2007).

Various molecular signaling and transcription factors regulate the differentiation of epithelial cells in airway development. For example, SOX2 plays a critical role in airway cell fate determination, and the loss of *Sox2* expression causes the failure of a generation of the club and ciliated cell fates (Que et al. 2009; Que et al. 2007; Tompkins et al. 2009). Meanwhile, Notch signaling is indispensable in regulating the balance of differentiation into the club and ciliated cells in developing airways. It is essential for club cell differentiation, and inhibition of Notch signaling leads to an increase of ciliated cells and a decline of club cells (Tsao et al. 2009). Otherwise, *FOXJ1* is an essential regulator for ciliated cell differentiation in airway developmental processes (Chen et al. 1998).

The progression of lung branching morphogenesis terminates upon the alveoli formation. Alveolar epithelial cells arise from *Sox9* and *Id2* double positive distal tips in the mouse developing lung (Fig. 2). Previously, lineage tracing of *Id2-*positive cells in mouse lung development indicated that those cells were multipotent before E13.5 and could generate both proximal and distal epithelial cell lineages, including neuroendocrine cells (Rawlins et al. 2009). In addition, the stem cell propensity of these tip cells declines at E16.5, which could only give rise to descendants of alveolar lineages. In the mouse, the alveolar epithelial cells mature just before birth, but the exact timing of AT1 and AT2 cell fate decision is still unknown.

Lineage tracing of *Id2*^+^ cells shows a progressive lineage-restricted model of alveolar epithelial cell fate determination, whereas the tip progenitors generate AT2 cells, then a subset of AT2 cells convert to AT1 cells (Rawlins et al. 2009). However, another study suggests the bi-potent alveolar progenitors, which is present at the tip and characterized by co-expression of some AT1 and AT2 marker genes, are able to give rise to both AT1 and AT2 cells in the late stage of lung development (Desai et al. 2014). On the contrary, a study combining lineage tracing and single-cell RNA sequencing reveals that the time of cell fate commitment of AT1 and AT2 cells is much earlier than previously thought (Frank et al. 2019). This process happened before canalicular stage rather than saccular stage as discussed formerly. In addition, there is also evidence that mechanical cues act as important regulators in alveolar cell fate commitment during alveolarization. The inhaled amniotic fluid through fetal breathing movements is important for AT1 cell differentiation (Li et al. 2018). Furthermore, a subgroup of alveolar progenitors is capable of protruding out of the airway tubes and orientating the mesenchyme. The cell protrusion process leads to enriched myosin in the apical region of protruded cells; this myosin protects these cells from being flattened by mechanical forces and governs their AT2 cell fate (Li et al. 2018).

### Application of single-cell RNA sequencing in the lung development program

Accompanied with the progression and extensive application of single-cell RNA sequencing in lung development, cellular composition and new cell types have been unbiased and robustly assessed in unprecedented detail. The scRNA-seq data covering the earliest lung development stage after endoderm specification is at E9.5. A comprehensive atlas study profiling 2 million individual cells of 61 mouse embryos from E9.5-E13.5 identified a group of lung epithelial cells within the epithelial lineages of collective organs (Cao et al. 2019). Another scRNA-seq data from E9.5 mouse embryo found 3 groups of cells related to the respiratory system, including *Nkx2-1*^+^/*Sox2*^+^ tracheal cells, *Nkx2-1*^+^/*Sox2*^*−*^ lung cells, and foregut-mesoderm derived cells (Han et al. 2020). Dong et al. analyzed 1916 cells of 8 different organs of mouse embryo from E9.5-E13.5 and clustered those cells associated with lung development into 3 distinct cells, including epithelial cells, mesoderm-derived cells, and hematopoietic cells (Dong et al. 2018). Epithelium from various organs presents obvious differences between early (E9.5) and later (E10.5-E11.5) development stages. Trajectory analysis indicated that lung epithelial cells are much similar to skin epithelial cells in the early developmental stage, but the lung epithelial cells exhibit organ-specific progressive patterns at the later development stage. More interestingly, at the earlier stage, the epithelial-mesenchymal transition state exists in multiple organs, including the lung. By contrast, the later lung development process did not present an epithelial-mesenchymal transition, suggesting that the transition program of this kind of cell may be finished between E12.0-E18.0 (Dong et al. 2018).

ScRNA-seq analysis of 198 single cells from 3 different mouse lung development stages (E14.0, E16.5, E18.5) identified 4 known epithelial cells, including club cells, ciliated cells, AT1 cells, and AT2 cells, with specific marker genes expression (Treutlein et al. 2014). What is noteworthy is that the fifth kind of cell was identified, which co-expresses AT1 and AT2 cell marker genes, such as *Ager/Pdpn* and *Sftpb/Sftpc*. Cell trajectory analysis demonstrated that this group of cells might dictate bi-potential alveolar cell properties (Treutlein et al. 2014). In addition, another scRNA-seq study of fetal lung across W11.5 to W21 identified 12 epithelial clusters, two of which were newly found as tip progenitor and secretory progenitor, respectively (Miller et al. 2020). The tip progenitor that co-expresses AT1 and AT2 marker genes were residing in the airway at W11.5 and distal lung at W15, consistent with previous findings in mice (Treutlein et al. 2014). However, another study indicated that these bi-potential cells contribute little to AT1 and AT2 cells (Frank et al. 2019). CreERT2 labeling of AT1 maker gene *Hopx* and AT2 maker gene *Sftpc* revealed that more than 80% of alveolar cell fate has been already committed before canalicular stage. In order to confirm the result of lineage tracing, the transcriptome information of 7106 individual *Nkx2-1*^+^ cells has been analyzed and identified 11 clusters, including epithelial cells, endothelial cells, and neuroendocrine cells. *Nkx2-1*^+^ epithelial compartments consist of 4 distinct alveolar cell types: AT1 cells, AT1 progenitors, AT2 cells, and AT2 progenitors. Only a few *Nkx2-1*^+^ cells simultaneously exhibit AT1 and AT2 cell marker gene expression. Trajectory analysis further validates that cell fate commitment of AT1 and AT2 cells has been determined before the canalicular stage rather than the saccular stage (Frank et al. 2019).

Single-cell transcriptome analysis of P1 mouse lung identified a transient cell simultaneously expressing AT1 and AT2 cell marker genes (Guo et al. 2019), in agreement with previous findings (Desai et al. 2014; Treutlein et al. 2014). Moreover, a small number of cells highly expressing *Sox2* were defined as the progenitors of club and ciliated cells. A recent study combining single-cell genomics and lineage tracing revealed a spatio-temporal pattern of activated cellular crosstalk during alveolar development and maturation (Zepp et al. 2021). This study established a single-cell lung development atlas including epithelium, endothelium, and mesenchyme from the embryonic stage to post-natal phage. Subsequent analysis of ligand-receptor interaction between each cellular compartment revealed that AT1 cells act as a signaling hub communicating with the nearby mesenchyme (Zepp et al. 2021). Mesenchymal progenitors are segregated into distinct cell types according to their different molecular signaling expressions, including *Acta2-*, *Pdgfrb-*, or *Wnt2*-expressing subsets. The progenitors of secondary crest myofibroblasts (SCMFs) were detected via scRNA-seq and lineage tracing, which present only during the earlier postnatal alveolarization phase of lung development, aligned with the progenitors of AT1 cell spatially and transcriptionally (Zepp et al. 2021). Compared to other mesenchymal cell lineages, SCMFs exhibit greater mechanical forces in vivo, driving the architectural formation of the alveolar niche. Chromatin accessibility and signaling pathway analysis of AT1 cell and SCMF at single-cell level suggested that *Foxa* and *Tead* are the upstream regulators of *Wnt* and *shh* that are secreted from AT1 cells, and the downstream regulated genes of *Shh* and *Wnt*, e.g., *Gli1* and *Tcf *are in open chromatin status of SCMFs. Accordingly, the specific deletion of *Shh* signaling in AT1 cells leads to the reduction of SCMFs and substantial simplification of the alveoli (Zepp et al. 2021).

## Stem cells in adult lung and regeneration after injury

### Stem cells in airways

Previous studies have indicated that epithelium within mouse and human airways has a low turnover rate during homeostasis. However, when the airway epithelium is injured, the resident stem cells in the airway will re-enter the cell cycle and re-initiate proliferation and differentiation, generating other epithelial cell lineages (Table 1).

**Table 1:** Summary of stem cells in adult lung

Stem cell	Markers	Regenerative Ability	Resident Region	Refs
Basal cells	TRP63, KRT5, NGFR	Basal cells are cable of populating proximal airway epithelium	Trachea and proximal airway in human, while trachea and main bronchi in mouse (Unlike in human, mouse small airways do not contain basal cells)	Hogan et al. 2014; Morrisey and Hogan 2010; Nikolić et al. 2018; Wu and Tang 2021; Zepp and Morrisey 2019
Club cells	SCGB1A1, SCGB3A2	Club cells represent powerful stem cell ability within mouse lung after injury, though lack of basal cells	Mouse airways within lung (Whether club cells within human lung also have similar stem cells capability still need to be validated)	Giangreco et al. 2002; Hong et al. 2001
LNEPs	SOX2, TRP63	LNEPs migrate from distal medium and small airways to the damaged alveolar region and form KRT5 pod, but lack of ability to generate alveolar cells	Alveoli	Kanegai et al. 2016; Kumar et al. 2011; Ray et al. 2016; Vaughan et al. 2015; Xi et al. 2017; Yang et al. 2018; Zuo et al. 2015
BASCs	SCGB1A1, SFTPC	BASCs can be stimulated at injured environments to differentiate into multiple cell lineages, including airway epithelial cells and alveolar cells	Broncho-alveolar junction (BADJ)	Kim et al. 2005; Lee et al. 2014; Liu et al. 2019; Salwig et al. 2019
RASCs	SCGB1A1 SCGB3A2	RASCs can convert into AT2 cells and this process is disrupted in COPD patients	Respiratory airways	Basil et al. 2022
AT2 cells (AT2^Axin2^)	SFTPC, ABCA3	A group of AT2 cells that respond to Wnt signaling can proliferate and differentiate into AT1 cells	Alveoli	Nabhan et al. 2018; Zacharias et al. 2018

#### Basal cells

Basal cells are now generally accepted as stem cells/progenitors of proximal airways. When airway epithelium is damaged or under physiological renewal, basal cells could proliferate and differentiate into other kinds of epithelial cells. More specifically, basal cells are a group of cells that intimately localize in the basal layer of the airway and specifically express *Trp63* (transformation-related protein 63), *Krt5* (keratin 5), and *Ngfr* (nerve growth factor receptor). Basal cells are distributed from the trachea to terminal bronchioles in humans, whereas they are mainly present in mouse trachea (Hogan et al. 2014; Morrisey and Hogan 2010; Nikolić et al. 2018; Wu and Tang 2021; Zepp and Morrisey 2019).

In normal status, the turnover rate of basal cells is low. However, such as exposure to sulfur dioxide, basal cells will be stimulated when airway epithelium is injured. In that condition, these cells start to proliferate to increase their cell number within the first 24 h and subsequently differentiate into club cells and ciliated cells. A number of studies have shown that Notch signaling is critical in regulating the differentiation of basal cells (preferentially acquiring a club cell fate). Inversely, loss of Notch signaling makes basal cells transit into a ciliated cell fate (Mori et al. 2015; Pardo-Saganta et al. 2015). Apart from Notch, there are multiple signaling inputs participating in this process, such as FGF10, p53, and Hippo signaling (Mahoney et al. 2014; McConnell et al. 2016; Volckaert et al. 2011). Recent findings combining scRNA-seq and lineage tracing demonstrated that the stem cell abilities of basal cells are different between homeostasis state and regeneration after acute injury of airway epithelium (Montoro et al. 2018). During homeostasis, basal cells are likely to produce club cells first, and then club cells trans-differentiate into a ciliated cell fate (Montoro et al. 2018; Rock et al. 2009). However, under an acute injury, basal cells can directly give rise to both club cell and ciliated cell lineages (Mou et al. 2016; Pardo-Saganta et al. 2015). It, therefore, hinted that there is considerable heterogeneity in basal cell populations: cells expressing NOTCH2 preferentially gain a club cell fate, while cells expressing MYB (a transcriptional activator) are likely biased to a ciliated lineage (Pardo-Saganta et al. 2015). This heterogeneity presumably endows basal cells with differentiation flexibility in response to airway damage.

#### Club cells

Although basal cells act as stem cells in proximal airways, holding the potential to give rise to other cell lineages during homeostasis and after injury, basal cells are scarcely distributed in airways within mouse lungs. In comparison, multiple club cells exist in mouse airways within the lung and have stem cell abilities in charge of regeneration after lung airway injury. For example, naphthalene treatment in mice causes club cells selectively ablated, while other cell types are mostly kept. In this model, rare survival club cells re-enter the cell cycle and repopulate the airways in a few weeks (Giangreco et al. 2002; Hong et al. 2001) due to lacking expression of the cytochrome CYP2F2, which is needed for naphthalene metabolic processing.

Since the basal cells are not frequently observed at every corner of the airways of the human lung, it is still uncertain whether club cells within human lungs also have similar stem cell capability. Recently, a study on COVID-19 patients indicated that plenty of proliferating cells in small airways (diameter < 0.5 mm) lack KRT5 expression (Fang et al. 2020). Thus, an exciting future direction will be identifying and comparing all kinds of resident stem cells/progenitors involved in airway regeneration after lung damage.

#### Lineage-negative epithelial progenitors (LNEPs)

In the acute lung injury model induced by bleomycin or influenza virus infection, a subset of cells with canonical basal cells markers expression, such as KRT5 and TRP63, could be detected in alveolar regions (Kanegai et al. 2016; Kumar et al. 2011; Ray et al. 2016; Vaughan et al. 2015; Xi et al. 2017; Yang et al. 2018; Zuo et al. 2015). Lineage tracing has shown that basal cell-like cells are marked by SOX2 and TRP63 rather than KRT5, indicating that the expression of KRT5 in those cells is specifically triggered by damage somehow (Ray et al. 2016; Yang et al. 2018). Therefore, this group of cells is named lineage-negative epithelial progenitors (LNEPs) (Vaughan et al. 2015; Zuo et al. 2015), and sometimes also called distal airway stem cells (Kumar et al. 2011).

The LNEPs could be flattened to cover severely damaged basement membranes of the alveolar region. Apart from that, their potential to differentiate into AT1 or AT2 epithelial cells is limited. These cells still keep in ‘airway-like cystic structures’ instead of forming the functional units of the alveolar region up to 200 days’ post-infection (Kanegai et al. 2016).

However, some studies demonstrated that there are signaling pathways important for LNEPs to trans-differentiate into alveolar epithelial cells (Ray et al. 2016; Vaughan et al. 2015; Xi et al. 2017). For example, Notch signaling is essential for the stimulation of KRT5 and TRP63 (Ray et al. 2016), but Notch suppression would drive LNEPs into AT2 progeny (Vaughan et al. 2015). In addition, the deletion of hypoxia-inducible factor 1 alpha (Hif1a) or persistent activation of Wnt signaling could also induce an AT2 cell fate transition (Xi et al. 2017).

#### Bronchioalveolar stem cells

There are small populations residing in mouse bronchoalveolar junction (BADJ), expressing both club cell marker gene SCGB1A1 and AT2 cell marker gene SFTPC (Kim et al. 2005; Lee et al. 2014). Those groups of cells are referred to as bronchioalveolar stem cells (BASCs) (Kim et al. 2005) due to their specialized localization and stem cell abilities. More recently, we have much more knowledge about these cells through lineage tracing. Two groups using different dual lineage tracing tools (Dre/Cre or split-Cre/split-tTA) have both revealed that bronchioalveolar stem cells could be stimulated in response to different injuries and differentiate into multiple epithelial cell lineages, including club cell lineage, ciliated cell lineage, AT1 epithelial cell lineage and AT2 cell lineage (Liu et al. 2019; Salwig et al. 2019). However, whether their traces could be found in human lungs is not known now.

#### Respiratory airway secretory cells

Recently, Basil identified a group of a cell population using scRNA-seq analysis of normal human distal lung tissue, which was named respiratory airway secretory cell (RASCs) for its unique location in the respiratory airway (Basil et al. 2022). Different from common secretory cells, RASCs express not only canonical marker *SCGB1A1* but also *SCGB3A2*. Surprisingly, RASCs can convert into AT2 cells when cultured in an alveolar medium, and this transition is unidirectional and partially regulated by Notch and Wnt signaling. But this transition process is disrupted in COPD patients or exposed to cigarette smoke constantly and associated with alternative AT2 state co-expressing *SCGB3A2* and AT2 maker *LAMP3* (Basil et al. 2022).

### Stem cells in alveoli

#### AT2 cells

Lineage tracing studies have established that AT2 cells can self-renew and differentiate into AT1 cells in the alveolar epithelium (Barkauskas et al. 2013; Rock et al. 2011) (Table 1). This stem-cell property is displayed both during homeostasis and after injury, such as lung regrowth after pneumonectomy and lung injury induced by bleomycin (Barkauskas et al. 2013). The remaining AT2 cells could re-enter the cell cycle and undergo rapid clone expansion if many AT2 cells were specifically ablated (Barkauskas et al. 2013). In various mouse lung injury models, lineage tracing indicated that AT2 cells can self-renew and convert to AT1 cells. Two independent studies suggested that Wnt signaling responsive AT2 cells (AT2^Axin2^) are dominated in alveolar stem cells (Nabhan et al. 2018; Zacharias et al. 2018). Influenza-mediated injury showed that those AT2^Axin2^ cells could proliferate around most of the damaged alveolar regions. Apart from that, they are also able to produce a considerable number of AT1 cells. Thus, those AT2^Axin2^ cells have been referred to as ‘alveolar epithelial progenitors’ (AEPs) (Zacharias et al. 2018). More importantly, due to their powerful stem cell abilities, AEPs are the primary force of alveolar regeneration after injury. According to the data on transcriptome and open chromatin structure, AEPs exhibit a unique transcriptional and epigenetic status compared with whole AT2 cells as a group (Zacharias et al. 2018). Additional data suggest that mesenchyme-derived signaling, such as Ffg7, Fgf10, and Wnt signaling, modulate the self-renewal of AEPs and differentiation into matured AT1 and AT2 cells (Nabhan et al. 2018; Zacharias et al. 2018). Subsequently, a single-cell molecular atlas of the human lung identified a subset of AT2 cells with increased Wnt signaling, which is homologous to the Wnt-active subpopulation of mouse AT2 cells (AEPs), could be alveolar stem cells in the human lung (Travaglini et al. 2020).

Some studies have demonstrated that mechanical tension is critical in regulating the proliferation and differentiation of AT2 cells (Liu et al. 2016). In the mouse pneumonectomy model, significantly decreased alveoli results in increased mechanical tension of the remaining alveolar epithelium (Liu et al. 2016; Wu et al. 2020). The increased force causes actin aggregation of actin in AT2 cells, then this process promotes both the nuclear transport of YFP and the subsequent YFP-related proliferation (Liu et al. 2016). YFP is a coactivator of transcription, acting as the mechanical tension effector of the nucleus to promote cell proliferation in many epithelial tissues (Dupont et al. 2011). Apart from that, increased mechanical tension accelerates the conversion of AT2 cells to AT1 cells as well.

Multiple studies of single-cell RNA sequencing have identified an AT2-AT1 cell state during the regeneration of the alveolar region (Choi et al. 2020; Jiang et al. 2020; Kobayashi et al. 2020; Riemondy et al. 2019; Strunz et al. 2020; Wu et al. 2020). For example, three different AT2 cell states were identified in a lipopolysaccharide-induced mouse alveolar injury model, including proliferating state, cell cycle arrested state, and transitional state (Riemondy et al. 2019). In the bleomycin-induced mouse alveolar injury model, a subset of AT2 cells exhibit significantly reduced classical AT2 maker genes expressions, such as *Cxcl15*, *Sftpa1,* and *Sftpc*, but do not express AT1 maker genes at an accelerated level. RNA velocity analysis revealed that this intermediate state is in a situation between conversion from AT2 cells to AT1 cells (Strunz et al. 2020).

AT2-AT1 cell states transiently express some molecular biomarkers, including *Cldn4*, *Sfn,* and *Krt8*, which could specifically identify this intermediate cell state. Lineage tracing indicated that AT2 cells at this intermediate state can differentiate into AT1 cells subsequently, which further confirms that it is necessary for AT2 cells to go through this intermediate state before transiting into AT1 cells (Jiang et al. 2020; Kobayashi et al. 2020; Riemondy et al. 2019; Strunz et al. 2020; Wu et al. 2020). Some recent findings show that AT2 cells were stuck at this intermediate state during the differentiation into AT1 cells in idiopathic pulmonary fibrosis (IPF) (Kobayashi et al. 2020; Strunz et al. 2020), supporting that the AT2 cells in IPF cannot differentiate into AT1 cells (Jiang et al. 2020; Wu et al. 2020). By combining with the application of some technologies, including the AT2 cell organoid culture system, scRNA-seq, and mouse models, one group found that cell senescence and P53 signaling are significantly enriched at this intermediated state (Kobayashi et al. 2020). Another group using scRNA-seq demonstrated that TGFβ signaling was activated in cell populations at that intermediate state (Wu et al. 2020). Besides that, the AT2-AT1 cell state was also found in patients with acute respiratory disease symptoms and diffuse alveolar damage via immunofluorescence staining (Chen et al. 2020; Strunz et al. 2020). Recently, scRNA-seq performed on human distal lungs and alveoli identified a unique epithelial transition state termed AT0 cell in primate lung regeneration and disease (Kadur Lakshminarasimha Murthy et al. 2022). This AT0 cell derived from the AT2 cell has the potential to differentiate into AT1 cells or terminal secretory cells cultured in vitro. But it prefers to convert into terminal secretory cells in severe pulmonary fibrosis, forming ‘bronchiolized regions’ (Kadur Lakshminarasimha Murthy et al. 2022).

Interestingly, a subgroup of AT1 cells expressing Hopx^+^ could be capable of proliferation and giving rise to Sftpc^+^ AT2 cells in rare instances after pneumonectomy (Jain et al. 2015). However, a recent study assessed the heterogeneity within the AT1 cell populations and found that the most mature AT1 cell subtype marked by Igfbp2 lacks differentiation capacity following pneumonectomy (the AT1 cell population consists of two distinct subtypes that differ in cell fate), pointing to the not-fully-resolved characteristic of AT1 cells. Hence, it is a prerequisite to make the physiological and pathological contributions of AT2 cells in human lung diseases much clearer.

## Limitations and future perspectives

The current knowledge about newly defined cell types and progenitor status is mainly based on scRNA-seq technology due to its powerful strength to dissect cell lineages and molecular changes during lung development and regeneration. However, the heterogeneity of the lung presents not only on a cellular but also anatomical level. For example, the formation of branched ductal tree-like and alveolar structures depends on plenty of molecular and cellular events, especially the intercellular interaction and microenvironment impact. Therefore, the spatial molecular dynamics of distinct cell types need to be further studied. Currently, methods of obtaining spatial gene expression patterns are mainly clustered into two groups. One is based on in situ hybridizations and imaging such as MERFISH (Moffitt and Zhuang 2016) and seqFISF^+^ (Eng et al. 2019), and the other relies on next generation sequence, including Geo-seq (Chen et al. 2017), Slide-seq (Rodriques et al. 2019), Visium, HDST (Vickovic et al. 2019), etc. With the integration of these methods with scRNA-seq, novel cell types and marker genes could be mapped back onto the tissue sections of interest by employing experimental validation or computational inference.

The new cell types defined in lung tissue currently mainly rely on gene expression. Therefore, the functional characterizing and underlying regulatory mechanism would be the next research effort. The single-cell measurements of the genome sequence, chromatin accessibility, DNA and histone modifications, small RNAs, and cell surface proteins have also empowered us to deepen our understanding of lung developmental and pathogenic mechanisms. The number of omics datasets describing lung development would be continuously growing, thus depicting lung developmental biology and lung regenerative medicine in more sensitive, accurate, and detailed ways.

There is a difference in the anatomy between mouse and human lungs. Obviously, the size of the human lung is larger than the mouse lung, with two left and three right lobes, compared to one left and four right lobes in the mouse. The airway branch generations of the human lung are also more than the mouse (17–21 in humans versus 13–17 in mice) (Irvin and Bates 2003). Additionally, human cartilaginous airways extend to segmental, intrapulmonary bronchi, whereas in mice, they end at the lobar, extra-pulmonary bronchi. Submucosal glands (SMGs) are present throughout the cartilaginous airways in human lungs but are restricted to the most proximal region of the trachea in mice. Importantly, mouse lungs lack respiratory bronchiolar epithelium in the narrowest airways around the entrance close to the alveoli. Notably, the unique structures in the human lung, such as terminal and respiratory bronchioles (TRBs), are involved in respiratory diseases, including idiopathic pulmonary fibrosis (IPF) (Verleden et al. 2020) and chronic obstructive pulmonary disease (COPD) (Tanabe et al. 2017). Therefore, while great strides have been made in characterizing the molecular events driving lung development in mice, it is still needed to make great progress in human lung studies, even though there are many challenges in sampling human tissue. Furthermore, the cross-species analyses between human and other model species should be performed to classify cell type homologies and diversifications. Recently, a human lung cell atlas identified a sub-group of AT2 cell population (Travaglini et al. 2020), homologous to the rare, Wnt signaling activated subpopulation of mouse AT2 cells (AT2^stem^), which could be alveolar stem cells of the human lung. But this need to be further clarified for the differences between human Wnt active AT2 cells and ‘bulk’ AT2 cells are not shared by AT2 ^stem^ cells in the mouse lung. Although there is profound evolutionary conservation of lung cell types between humans and mice, the extensive plasticity of cell types and cell-type-specific gene expression during lung evolution still exist. There are four types of expression divergence: 1) genes express in the same human and mouse lung cell type 2) genes show simple gain or loss 3) expression changes involved in extra cell types 4) changes present a switch in expression from one cell type to another (Travaglini et al. 2020). Therefore, additional studies are required to elucidate the molecular mechanism underlying those gene expression differences across species and organ evolution.

## Conclusions

Lung diseases are the primary cause of death worldwide. Multiple cell types within adult human lungs express lung disease genes and virus receptors encoding genes, which are associated with the occurrence of lung diseases and infection of respiratory viruses (Travaglini et al. 2020). Recent progress in single-cell genomic and transcriptomic analysis with high throughput and resolution helps us establish a human cell lung atlas, serving as a precise reference for human lung in health and disease (Schiller et al. 2019). Moreover, a consortium of various research groups is funding an effort to construct a molecular atlas of lung development (LungMAP: https://lungmap.net/) for a better understanding of the process which regulates the lung development program, especially alveologenesis (Ardini-Poleske et al. 2017). Elucidating the orchestration of lung development is crucial for recovering the causes behind a wide range of chronic and acute respiratory diseases. For example, the exploration of lung branching morphogenesis and alveologenesis may allow us to find a way to treat lung diseases and regenerate injured lungs.

**Fig. 3:**
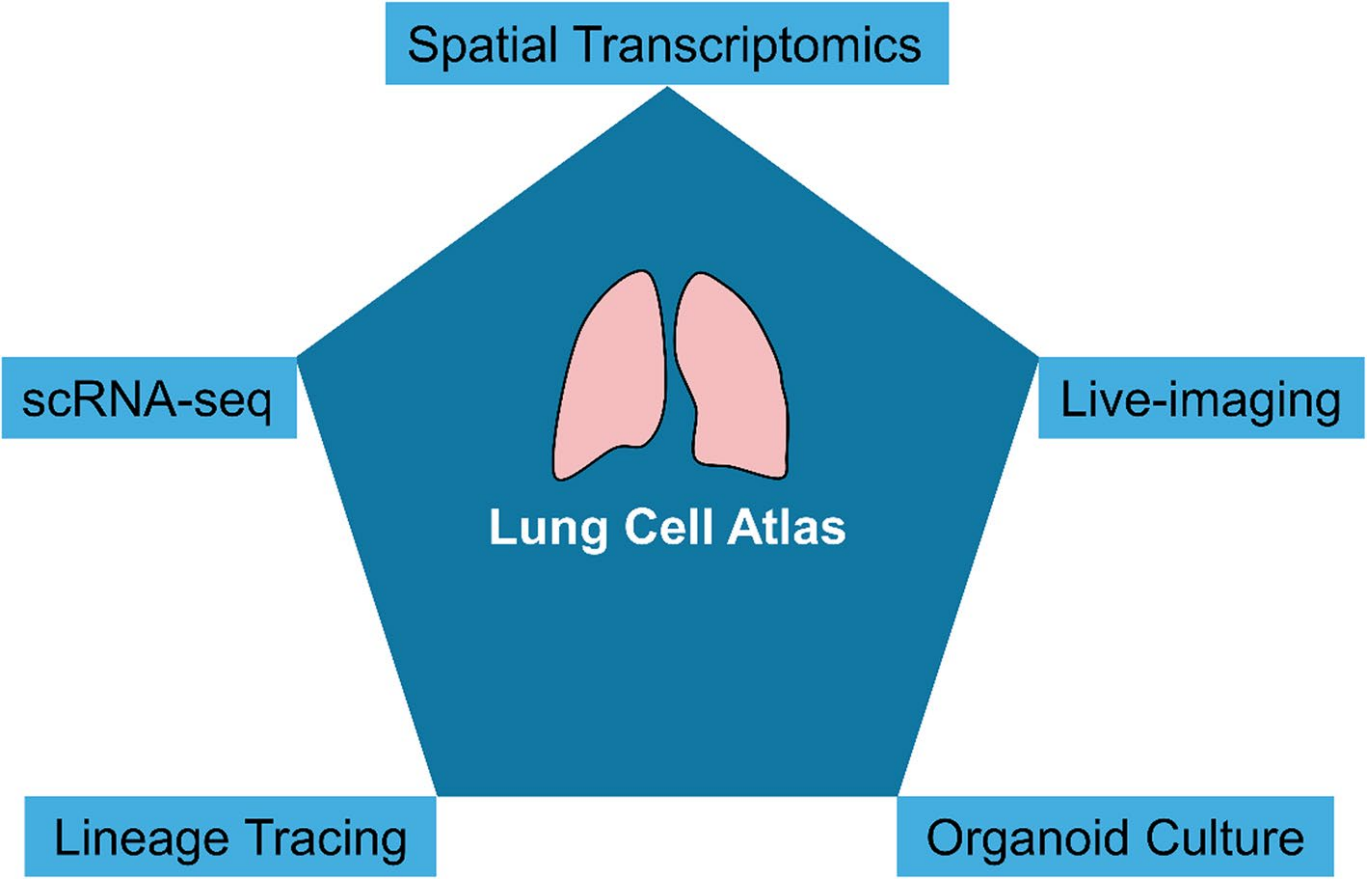
Technologies that enhance the lung cell atlas. The spatial transcriptomics, scRNA-seq, live-imaging, lineage tracing and organoid culture system will strengthen the reference cell atlas with high spatiotemporal resolution to facilitate the molecular profiling of new function cell types during mammalian lung development and regeneration

Although scRNA-seq has provided many new insights into lung development and regeneration, the use of a multimodal approach is increasingly demanded due to the complexity of cellular composition and spatial heterogeneity in the lung tissue. In this regard, powerful technologies, including spatial transcriptomics, lineage tracing, lung organoid culture, and imaging techniques coupled with scRNA-seq could make the molecular atlas of the lung and its development process more precise and complete (Fig. 3). New genetic mouse models based on the engagement of independent DNA recombinases can be implemented to trace rare cells which are identified by distinct markers. Establishment of lung organoid culture systems could be used to test specific signaling molecules as well as the supporting cells acting as different niches for the roles they played in regulating proliferation and differentiation of stem cells/progenitors. Furthermore, the demonstration of morphological changes in lung cells during development and regeneration highly relies on progress in imaging techniques, particularly on higher resolution and live-imaging. Eventually, all these techniques would be more commonly used in the near future to characterize the undefined cell types and progenitor status in the developing and adult lung, each of which would generate rich data. However, it is still necessary to integrate those techniques together to comprehensively dissect the regeneration ability of the lung from distinct aspects to provide a deeper understanding of cellular hierarchy and the presence of a spectrum of transitional molecular phenotypes along a trajectory of possible cell states, and cell populations and their precursors and progeny that dynamically change over time, in space and in health or disease.

## Data Availability

Not applicable.

## Abbreviations

AEPs: Deemed alveolar epithelial progenitors
AT1 cells: Alveolar type 1 cells
AT2 cells: Alveolar type 2 cells
ARDS: Acute respiratory distress syndrome
BADJ: Bronchoalveolar junction
BASCs: Bronchioalveolar stem cells
BPD: Bronchopulmonary dysplasia
COPD: Chronic obstructive pulmonary disease
COVID-19: Corona virus disease 2019
IPF: Idiopathic pulmonary fibrosis
LNEPs: Lineage negative epithelial progenitors
PNECs: Pulmonary neuroendocrine cells
RDS: Respiratory distress syndrome
scRNA-seq: Single-cell RNA sequencing
SARS-CoV-2: Severe acute respiratory syndrome coronavirus 2
SCMFs: Secondary crest myofibroblasts
SMC: Smooth muscle cell
SMGs: Submucosal glands
